# Applying of Pulsed Electromagnetic Processing of Melts in Laboratory and Industrial Conditions

**DOI:** 10.3390/ma11060954

**Published:** 2018-06-05

**Authors:** Valeriy Krymsky, Nataliya Shaburova

**Affiliations:** Polytechnical Institute, South Ural State University, Lenin Avenue, 76, Chelyabinsk 454080, Russia

**Keywords:** nanosecond electromagnetic pulses, aluminum alloy, microstructure and properties, melt treatment out in a furnace, ultrasonic treatment of metals

## Abstract

The use of various external influences to influence metal melts (vibration, ultrasound, etc.) is a known method of changing the structure and properties of metals and alloys. In the overwhelming majority, all methods of external action on melts cause grinding of the metal structure, which leads to an increase in strength characteristics. The paper considers a new method of external physical action on molten metal, namely, electromagnetic pulses. Work on the investigation of the impulse effect on metal melts is conducted in two laboratories: in Chelyabinsk (the laboratory of Professor Krymsky V.V.) and in Khabarovsk (in the laboratory of Ri Josen). If at the beginning only small masses of metal were processed in the laboratory, now the work is at the industrial level. Masses of processed metal reach 2 tons. The article summarizes and structures the results of the conducted studies on the effect on nonferrous metal melts with powerful electromagnetic pulses. General regularities of such influence on the structure and properties of the metal are established. The results of such effects on pure metals (aluminum, zinc) and on aluminum alloys are provided. It is established that impulse processing contributes to a decrease in the porosity of castings, an increase in metal density, and a decrease in electrical resistivity. Also, in pulsed processing, a grinding of the metal grains occurs, an increase in the solubility of the main components in the alpha phase, and changes for the eutectic in the structure. An interesting fact is the simultaneous increase in the properties of the strength and plasticity of the metal.

## 1. Introduction

Currently, there are many methods of external influence on melts with which it is possible to change cast metal structure. All of them can be divided into two groups: chemical methods and physical methods. Chemical methods (alloying, modifying, refining) are associated with a change of the chemical composition of metal. Physical methods (thermal, barometric, gravitational, mechanical, electromagnetic, high-energy) are based on the energy interaction of the metal with the environment. The most common methods of external physical impact on metal melts are electromagnetic stirring, vibration, and ultrasonic treatment. Each of these methods has its advantages and disadvantages, but all are capable of changing the structure and properties of cast metal in the right direction. The electromagnetic pulses (EMP) described in this article were originally used to create radar stations with high resolution and had not the slightest relation to metallurgy. The parameters of the pulses used were selected depending on the tasks to be solved. Over time, an ideal range of EMP pulse characteristics was developed: 0.1–1 ns duration, pulse repetition frequency 1 kHz. The range of the station and the depth of the detected objects were determined by the amplitude of the pulse.

In the future, the scope of EMP will be significantly expanded. The author of these new directions of using EMP can rightfully be called V.V. Krymskii. He first proposed the use of powerful nanosecond electromagnetic pulses (NEMP) for metal melts [1,2]. Znamensky L.G. received the first positive results for casting alloys [3]. Shaburova N.A. systematized the results of pulse treatment of ferrous and non ferrous metals [4,5]. Balakirev V.F. et al developed a theory of NEMP action on metal melts [6,7].

At present, two laboratories are working on the investigation of the impulse action on metal melts: in Chelyabinsk (laboratory of Professor Krymskii V.V.) and in Khabarovsk (in the laboratory of Ri Josen). If at the beginning only small masses of metal were processed in the laboratory, now the work is at the industrial level. Masses of processed metal reach 2 tons. This article summarizes and structures the results of the conducted studies on the effect on nonferrous metal melts with powerful electromagnetic pulses. General regularities of such influence on the structure and properties of the metal are established.

## 2. Experiment

### 2.1. Material and Equipment

Pure granulated aluminum of the following composition: 99.8 wt % Al, 0.04 wt % Si, 0.06 wt % Fe, 0.0015 wt % Cu, and 0.018 wt % Zn was used in our experiment. The weight of the base metal was 0.35 kg.

Zinc of the following chemical composition was investigated: no less than 99.98 wt % Zn; 0.005 wt % Pb; <0.001 wt % Cd; 0.0041 wt % Fe; 0.001 wt % Cu; 0.001 wt % Sn; <0.001 wt % Sb; <0.0005 wt % As.

AlSi7Mg, AlSi12 aluminum alloy of the Al-Si system was selected as a material for study. The chemical composition of AlSi7Mg alloy is: 6.4 wt % Si, 0.06 wt % Cu, 0.01 wt % Mn, 0.27 wt % Mg, 0.02 wt % Ti, 0.04 wt % Zn, 0.27 wt % Fe, and 92.9 wt % Al. The chemical composition of AlSi12 alloy complies with GOST 1583-93.

The study of aluminum alloys was conducted under experimental conditions (300 g and 3 kg of silumin treated) and under industrial conditions (120 kg of AlSi12 silumin treated).

**Figure 1:**
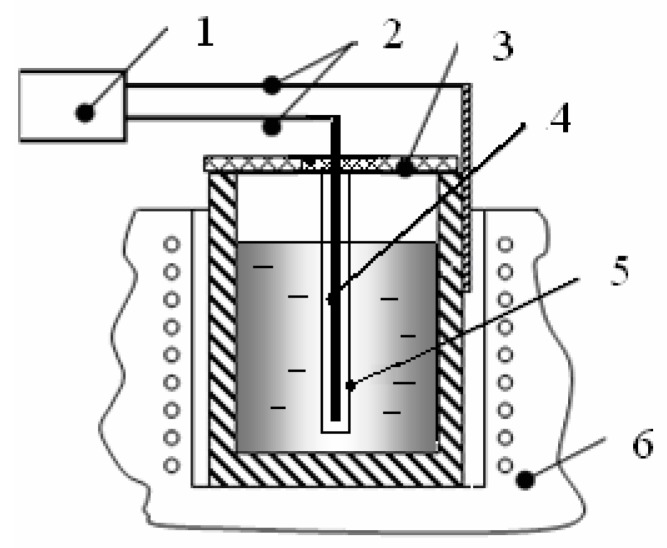
The installation diagram for electromagnetic pulse (EMP) treatment of metal melts: 1—EMP generator; 2—wires; 3—asbestos cover; 4—emitter; 5—protective quartz tube; 6—shaft resistance furnace.

The installation diagram for pulse electromagnetic treatment of the metal melt is shown in Figure 1. The metal was melted in a shaft resistance furnace. Generator (1) creates unipolar pulses: pulse duration—1 ns, amplitude—10 kV, pulse repetition frequency—1 kHz. One contact of the generator was closed on the graphite crucible, the second was connected to a copper radiator (4) and immersed in the melt. To protect the radiator from its contact with the metal melt, a quartz tube (5) was used.

### 2.2. Experimental Procedures

Two melting procedures were conducted in the identical time-temperature conditions. In one case, the metal was melted and kept for homogenization at a constant temperature. Then the furnace was turned off and the electromagnetic pulse radiator was immersed into the melt. The treatment was carried out for 15 min. Then the metal was cast. In the second case, the metal was kept in the turned off furnace for 15 min and then it was cast. The beginning treatment temperature and the casting temperature were controlled in both cases.

Laboratory and industrial experimental melting of aluminum alloys were carried out according to the following regimes. Laboratory experiments on the effect of EMP treatment time on AlSi7MgFe silumin properties were carried out by a team of our colleagues under the leadership of Ri Josen at Pacific State University.

The mass of the treated metal was 250–300 g. The melting was carried out in a graphite crucible of a “Parabaloid-4” installation (an installation for a complex study of physical properties of melts by the penetrating *g*-radiation method [8]). The temperature of the metal was determined by a tungsten-rhenium thermocouple. The metal was overheated to 900 °C then EMP-treated for 5, 10, 15, 20, and 25 min. Based on the obtained intensities of penetrating *g*-radiation, various characteristics of the metal were determined.

Laboratory experiments on the EMP treatment of metal with a mass of 3 kg were conducted at South Ural State University (Chelyabinsk). The metal (AlSi7Mg, AlSi12 alloys) was overheated to 780 °C, and then calcined cryolite flux amounting to 1% of the charge mass was injected. The melt was thoroughly mixed and held for 15 min. After that the slag was removed. Then the alloy was held in the turned off furnace for another 15 min and a half was poured into sand molds. The other half of the melt was re-treated in a similar way, but the melt in the furnace was treated with electromagnetic pulses (EMP) during the last 15 min.

The industrial experiment was conducted according to the same procedure. The mass of the treated AlSi12 melt was 120 kg, the first time in the world a silumin sample of this mass has been treated. The exposure time was limited to 10 min, because the melt started to heat up intensively near the EMP generator. 

The effect of EMP on zinc was carried out under industrial conditions in the manufacture of ball anodes. The weight of the processed metal is 300 kg.

### 2.3. Experimental Methods

Samples for metallographic analysis and mechanical testing were cut out from the obtained ingots. The microstructure was investigated with an Axio Observer.D1m optical microscope (Zeiss, Pleasanton, CA, USA) and a JEOL JSM-6460LV scanning electron microscope (Jeol, Tokyo, Japan), with energy dispersion spectrometer Oxford INCA X max 80 f (Oxford Inca, Oxford, UK) for elemental analysis. Mechanical characteristics were determined during tensile tests; hardness was measured by the Brinell hardness tester (Tochpribor, Ivanovo, Russia) and a micro hardness tester FM-800 (Future Tech Kanagawa, Japan).

The porosity of the samples was analyzed using a Neophot 21 optic metallurgical microscope (Carl Zeiss, Pleasanton, CA, USA) at 100-fold magnification. The comparison of porosity in the sections of the casts having one sample (140 mm^2^) cut from identical locations of an ingot were made.

Specific electrical resistivity was measured using a DML-48 Kelvin bridge in the cylindrical samples with a working length of 68 mm and a diameter of 4 mm (Zapadpribor, Moskow, Russia). The measurement accuracy was ±2 × 10^−4^ mcOhm·m.

To determine the mechanical characteristics, no fewer than five samples were tested. The values given in the work are the average arithmetic values for a series of samples.

## 3. Results and Discussion

### 3.1. Electric Pulse Processing of Pure Aluminum

The characteristics of the aluminum ingot processed according to standard procedure and processed by EMP were compared.

Study of the templates of pure aluminum ingots made it possible to outline several differences in the microstructure of the base metal and the EMP preprocessed metal. The structure of the longitudinal template of the aluminum ingot, not processed in the melted state, contains three distinct crystallization areas: the area of small equiaxed crystals at the walls of the casting form, the dominating area of fringe crystals, and the axial area of equiaxed crystals.

The template of the EMP processed aluminum ingot contains only two areas: the prevailing area of fringe crystals and the feebly-marked area of equiaxed crystals in the axial part of the ingot. Fringe crystals in the central parts of both ingots are arranged almost perpendicular to the axis of the ingot. Both samples have average linear grain sizes symmetrical to the ingot axis and perpendicular to the ingot axis. The following correlation of the average areas of the macrograins by the height of the ingots is characteristic: in the upper third portion of the ingots the average grain areas comprise 3.8 and 5.5 mm^2^; in the central portion: 4.8 and 3.4 mm^2^; and in the lower portion of the ingots: 3.3 and 4.4 mm^2^ respectively for raw and EMP processed metal.

The porosity of the metal ingots differs (on the average—143 and 166 pcs in the raw and EMP processed metal accordingly). As for the size spectrum of the pores, in the raw metal varied from 50 to 450 μm, while in the processed metal it varied from 100 to 250 μm.

The density of the metal determined through hydrostatic weighing was 2.682 and 2.695 g/cm^3^, accordingly. 

A Brinell hardness tester was used to determine the hardness of the samples. The measurement of the hardness of the aluminum samples has shown that the hardness of the raw metal is 17 HB, and the processed metal—23 HB.

Comparison of the values of specific electrical resistivity of the pure aluminum samples has shown that after the EMP exposure the electrical resistivity is decreased from 0.0277 Ohm·m to 0.0273 Ohm·m.

### 3.2. EMP Influence on Zinc

Molten zinc was exposed to EMP processing for 1.5 h at the initial temperature of the hot melt of 450 °C. Then the metal was put in to the 711-B-09 apparatus for pressure casting. Eight anode balls were cast simultaneously.

The comparative weighing of the anode balls cast by standard procedure and with EMP has shown that the weight of the anodes cast under the experimental procedure is 15–20% more (0.37 and 0.45 kg more on average, respectively). At the same time, all the anodes processed with the standard procedure had pores in the central portion.

Study of the templates has shown that in the EMP processed metal the sizes of pores considerably decrease (from 8–15 mm to 4–7 mm) and there is absolutely no sinkhole.

**Figure 2:**
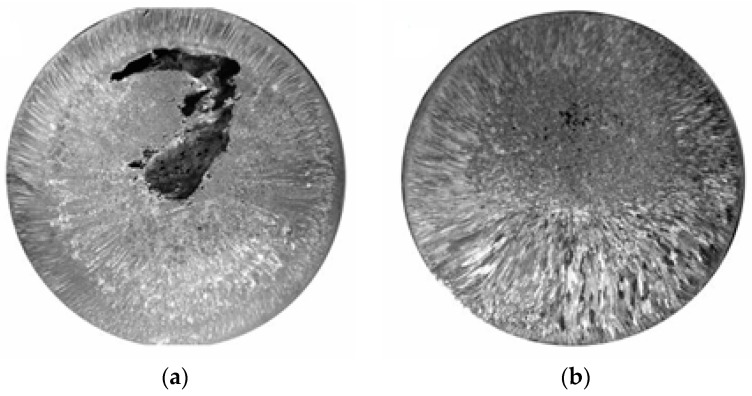
Macrostructure of the templates of pure zinc: (**a**) sample for comparison, (**b**) EMP processed sample.

Pictures of the macrostructures of zinc anodes are shown in Figure 2. The shots demonstrate considerable differences in the macrostructure. Thus, in the structure of the raw metal there is a 4–5 mm area at the surface with disperse fringe crystals of the average thickness of 0.1–0.15 mm. Large equiaxed crystals with the diameter of 1–1.5 mm prevail in the central part of the cast.

The processed metal also has two crystallization areas. Yet the area of fringe subcrystals has the width of about 9–11 mm, and the crystal sizes based on the results of the measurements by the random linear intercept method exceeded the crystal sizes of the raw metal 1.5–2 times. In the upper third part of the ingot, within the area of equiaxed crystals there are visible single dispersed pores with the size of 0.1 mm.

Study of fractures using a scanning electron microscope have shown that in both cases the metal has a fragile intercrystalline fracture.

The influence of EMP irradiation on the process of zincing rods for suspended insulators has been also studied. A 5140H steel rod was used. Zn0-A zinc alloy was exposed to irradiation in the steel hot zincing bath, 1200 × 800 × 800 mm. The weight of the irradiated metal was 3000 kg. Irradiation was performed over 4 h. In the course of irradiation, the temperature was maintained within 530 ± 20 °C by an automatic temperature controller. The irradiator in the form of a brass tube in a quartz tube was placed in the center of the bath.

Two test batches of rods made of unirradiated zinc (558 pcs.) and irradiated zinc (909 pcs.) were zinced. The thickness of the zinc coating in both batches was assessed in three sections of the rod: in the bowl, flare, and core. The average thickness of the coating in the batch zinced by the unirradiated metal comprised 117.6 μm, in the batch zinced by the irradiated metal—106.7 μm under the identical conditions of the zincing process. The maximum coating thickness variation in the batch of the unirradiated metal was 43.5 μm, in the batch of the irradiated metal—32.6 μm. The mechanical breaking load of the zinc coating of the unirradiated metal comprised 161 kN, of the irradiated metal—167 kN.

The results of EMP influence on molten metals are presented in the works [9,10].

### 3.3. Results of the Laboratory Experiments AlSi7MgFe

We studied how the duration of electromagnetic pulse treatment influenced the properties of liquid and crystallized AlSi7MgFe silumin and obtained the following results. When the treatment time increased, the melt crystallization temperature and the eutectic formation temperature increased by 30 and 10 °C, respectively. The degree of compaction and the thermal compression coefficient also increase linearly. The other characteristics, such as density, hardness, corrosion resistance, and heat resistance vary according to extremal relations. These properties reached their maximum when the pulse treatment lasted 15 min. Structurally, the size of dendritic cells varies according to the extremal relation when treatment duration increased; their minimum size was observed when the treatment lasted 10 min. When treatment time did not exceed 10 min the content of silicon in the *a*-solid solution reduced. Upon further irradiation it increased again. The particles of eutectic silicon became dispersed and compact [6].

AlSi7MgFe silumin samples of 3 kg each, which were casted under laboratory conditions, were compared to reveal differences in their microstructure. Above all, it should be noted that the EMP-treated metal cast lacked porosity. Its silicon phase of eutectic origin was more dispersed, it had the shape of needles with an average length of 25–30 μm and decreased from 30–35 to 10–15% of the structure, whereas the siliceous eutectic phase of the original metal had a rounded shape with average linear dimensions of 5–10 μm. Due to the decrease in eutectic discharges along the boundaries of the dendritic cells the α-phase dendrites slightly increased in size from 66 to 85 μm (the linear dimensions were determined by the random linear intercept method) and became more equiaxial. In addition, the microhardness of the α-solid solution increased by 10–15% (the hardness of the α-solid solution was 75 HV for the EMP-treated metal), this fact indicated that it was more alloyed. 

The increased solubility of the elements was also confirmed by studies carried out using the scanning electron microscope (SEM). According to the obtained data the maximum silicon concentration in the α-phase of the original metal reached 1.36–1.66 wt %, while that of the EMP-treated metal—1.48–1.80 wt % (with an absolute error of 0.04).

The hardness of cast samples of Al-Si alloys was measured; the melt that was pre-treated with EMP has shown an increase in hardness. For original samples the hardness was 51 HB, for the experimental—63 HB. The tensile strength was 170 MPa and 210 MPa, respectively, and the unit elongation was 4.8 and 18.4% for the original and the EMP-treated metal, respectively [3]. 

### 3.4. Results of the AlSi12 Industrial Experiment

**Figure 3:**
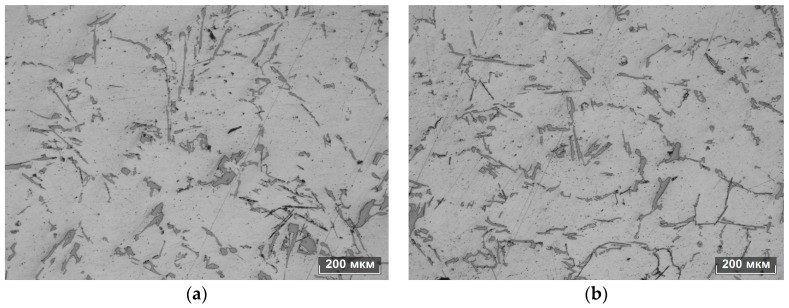
Microstructure of the original (**a**) and industrially treated (**b**) in AlSi12 alloys.

Although the total porosity of the original and treated AlSi12 alloy samples was the same (1 point in accordance with GOST 1583-93), the treated samples lacked blowholes and the friability in the cast centers halved in size (compared to the untreated metal). The comparative analysis of industrial melt samples showed the following. Both samples had a dendritic structure, their phase composition did not differ from the samples of laboratory experiments (Figure 3). The average dendritic cell area of the original sample was 0.02 mm^2^ (790 dendritic cells were measured); the cell anisotropy coefficient was 1.05. The average dendrite cell area of the EMP-treated metal was 0.03 mm^2^; the anisotropy coefficient was 0.96. The fraction of eutectic silicon in the original sample was 7%, in the treated sample the fraction of eutectic silicon was 9%. SEM studies showed that the average solubility of silicon in the *a*-solid solution of the original sample was 1.45 wt %, and in the treated sample—1.6 wt %. The microhardness of the original sample alpha phase was 83 HV, and that of the treated one—97 HV. The ultimate resistance and unit elongation of the original and treated samples after T5 heat treatment were: 292 and 282 MPa, and 4% and 2.4%, respectively.

### 3.5. Effect of Electromagnetic Pulse Treatment on AlSi7Mg

According to the equilibrium phase diagram the structure of hypoeutectic alloys of Al-Si system consists of α-phase precipitates and eutectic. Some intermetallic phases are also present in the alloys under consideration.

**Figure 4:**
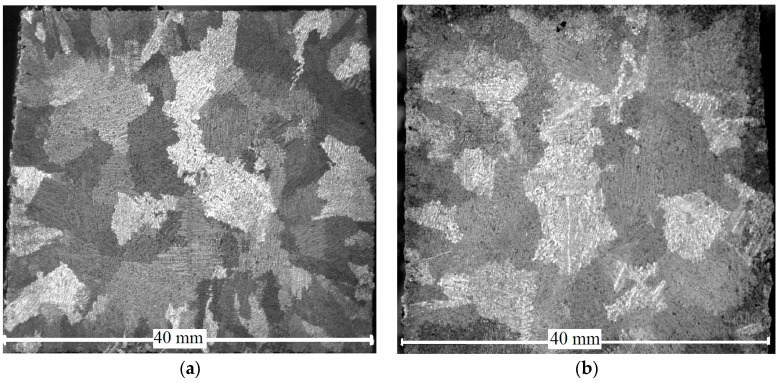
Macrostructure in AlSi7Mg alloy: (**a**) not treated by electromagnetic pulses; (**b**) treated by electromagnetic pulses.

Comparison of the microstructure of the samples, subjected to pulse electromagnetic treatment in liquid state and the samples, cast by conventional technology, helped to reveal some differences. The macrostructure of the crosscut of templates of AlSi7Mg alloy ingots is presented in Figure 4.

**Figure 5:**
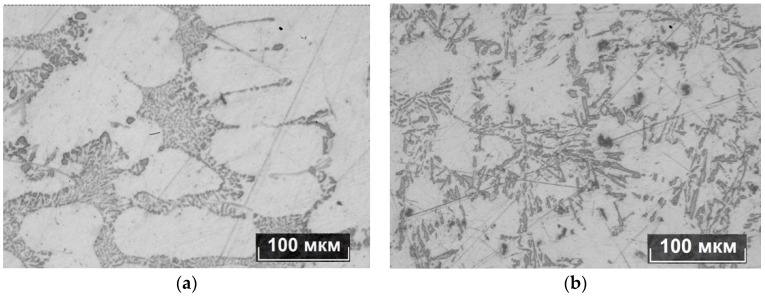
Microstructure of as-cast AlSi7Mg alloy: (**a**) not treated by electromagnetic pulses; (**b**) treated by electromagnetic pulses.

The template of untreated metal (Figure 4a) has two zones of crystallization: a zone of columnar crystals, located on the border with the mold and the zone of large equiaxic grains, located in the central part of the ingot. On the template of the metal treated with pulses (Figure 4b) the area of columnar crystals is less pronounced, rounded grains with the size of about 0.5–1 cm dominate. The observed crystallization structure indicates the uniform crystallization of solid phases throughout the volume of the treated metal. The microstructure of AlSi7Mg alloy was analyzed as well (Figure 5).

It is known that under equilibrium conditions with slow cooling of silumins castings that, besides the α-solid solution, irregular eutectic is formed: α-solid solution and silicon crystals as well as lamellar egesta, which is an AlSiFe compound, are formed. The primary α-phase in both samples has a dendrite shape. After electromagnetic treatment, the amount of eutectic in the structure decreases from 30–35% to 10–15%. The particles of eutectic silicon take the form of needles with a length of up to 25–30 μm. Silicon eutectic phase for untreated metal is dispersed with average linear dimensions up to 5–10 μm. By reducing the proportion of eutectic precipitates on the boundaries of dendrite cells, α-phase dendrites slightly increase their size from 66 to 85 μm (determination of the linear dimensions was performed by random linear intercept method) and become more rounded.

The microhardness of α-phase grains in the sample treated by electromagnetic pulses increased by 10–15% and reached 750 MPa. A reduction of the amount of eutectic and an increase of α-phase microhardness indicate a greater silicon content in this phase. The worsened etching of the treated metal is an indirect confirmation of the improved solution of elements in the α-phase.

Study of the composition of phases indicates an increase of silicon content. According to the obtained data the maximum concentration of Si in the α-phase of untreated metal reached 1.36–1.66 wt %. While in the treated metal—1.48–1.80 wt % (with an absolute error of 0.04).

Measurement of the hardness of the cast samples showed that pretreatment of the melt by electromagnetic pulses enhances hardness. Untreated samples have a hardness of 51 HB, treated samples—63 HB. In other words, hardness increases by more than 20%. Mechanical properties of the metal were determined during tensile tests. Tensile strength was 142.5 MPa and 166.8 MPa, elongation—4.5% and 5.6%, respectively, for untreated and treated metal.

### 3.6. Discussion

The results obtained by treating molten metal with electromagnetic pulses, demonstrate the influence of this treatment on the formation of the grain structure, composition and morphology of phase separation, and the mechanical properties of the metal.

The mechanism of such effects is not clear. However, by comparing the obtained results with the data of different types of external influence on the melt it became possible to find some similarities. The closest results are obtained from ultrasonic treatment of melts [10,11,12,13,14].

In the vast majority of works on ultrasonic treatment of aluminum, magnesium alloys and steels it is shown that the form of precipitates of the primary α-phase changes from dendritic to rosette and rounded.

Zhang L. et al [13] showed that ultrasonic treatment of metals with the power of 4 kW during crystallization facilitates the grinding of the macrograin of the metal from 1600 μm to 100 μm for 0.35 kg of Al-5 wt % Si alloy. Morphology of the α-phase grains changes to more rounded. The particles of the eutectic silicon acquire the same coarse shape as in electromagnetic treatment. These authors showed coarsening of eutectic in the Al-11 wt % Si and Al-17 wt % Si alloys.

Similarly, in [15], ultrasound treatment with the power of 1.5 kW and 20 kHz at temperatures close to crystallization lead to the coarsening of eutectic silicon. At the same time, the results of the work [14] on ultrasonic treatment at a power of 1.2 kW on 0.4 kg of AlSi9Cu alloy indicate a decrease of interlamellar distance and a size reduction of eutectic silicon particles. Other authors [15,16,17,18,19] note a similar influence of ultrasonic treatment for steels and nonferrous alloys in other systems.

Conflicting data from these and other authors on the changes in the morphology of eutectic silicon particles can be explained by the choice of temperature of exposure. When exposure occurs at the early stages of crystallization, the reduction of silicon particle size occurs as a result of cavitation. At lower temperatures, the solidified melt prevents the spread of cavitation flows, preventing the fragmentation of phases. Ultrasonic energy input into the melt, contributes to its heating, and thus changes the growth conditions of the eutectic phases, contributing to its coarsening. 

The increase of the content of the main alloying elements at the primary α-phase under the ultrasonic influence is mentioned in [14]. Increased strength and ductility of the material under ultrasonic treatment at 600 W and 19.5 kHz frequency, despite continued presence of coarse needle-shaped eutectic is mentioned in [2]. In the papers mentioned above a simultaneous increase in ultimate strength and elongation is observed alongside the grain size reduction and eutectic.

## 4. Conclusions

Comparative analysis of the results of ultrasonic and electromagnetic influence on smelt suggests similar mechanisms of their effect. To confirm this hypothesis, it is necessary to complete mathematical calculations. However, even now it is possible to say that despite the physical impossibility of propagation of electromagnetic pulses in smelt, their effect is a result of acoustic oscillations near the radiator. These oscillations contribute to the processes in a similar way to those that occur during ultrasonic treatment that is cavitation and sound pressure.

The advantage of the considered method of influence on metal melts lies in the fact that without changing the chemical composition of the metal, its mechanical properties can be changed in the desired direction.

## References

[1] Krymskiy V.V. (2001). Nanosecond Electromagnetic Pulses and Their Application.

[2] Balakirev V.F., Krymsky V.V., Kulakov B.A., Ri Khosen (2009). Electro-Impulse Nanotechnologies.

[3] Znamensky L.G., Krymsky V.V., Kulakov B.A. (2003). Electropulse Nanotechnology in Casting Processes.

[4] Shaburova N.A. (2011). Processing ofMetals and Alloys with Nanosecond Electromagnetic Pulses.

[5] Shaburova N.A. (2015). Changes in metal properties after thermal and electric impulse processing. Mater. Sci. Eng..

[6] Balakirev V.F., Krymsky V.V., Shaburova N.A. (2012). Nanopulse Technologies.

[7] Balakirev V.F., Krymsky V.V., Ri H., Shaburova N.A. (2014). Electric Pulse Treatment of Metal Melts.

[8] Ri E.H., Ri K., Dorofeev S.V., Yakimov V.I. (2008). The Influence of Irradiation of Liquid Phase by Nanosecond Electromagnetic Impulses for its Structure, Processes of Crystallization, Structure Formation and Properties of Casting Alloys.

[9] Shaburova N.A., Krymsky V.V. (2016). Electropulse Machining of Metals. Solid State Phenom..

[10] Eskin G.I. (1988). Ultrasonic Treatment of Molten Aluminum.

[11] Zhang L., Eskin D., Miroux A., Katgerman L., Suarez C.E. (2012). Formation of microstructure in al-si alloys under 293 ultrasonic melt treatment. Light Metals 2012.

[12] Puga H., Costa S., Barbosa J., Ribeiro S., Prokic M. (2011). Influence of ultrasonic melt treatment on microstructure 296 and mechanical properties of AlSi9Cu3 alloy. J. Mater. Process. Technol..

[13] Lin C., Wu S., Lü S., An P., Wan L. (2013). Microstructure and mechanical properties of rheo-diecast hypereutectic Al–Si alloy with 2%Fe assisted with ultrasonic vibration process. J. Alloys Compd..

[14] Li X.T., Li T.J., Li X.-M., Jin J.-Z. (2006). Study of ultrasonic melt treatment on the quality of horizontal continuously cast Al–1%Si alloy. Ultrason. Sonochem..

[15] Khalifa W., Tsunekawa Y., Okumiy M. (2010). Effect of ultrasonic treatment on the Fe-intermetallic phases in ADC12 die cast alloy. J. Mater. Process. Technol..

[16] Liu Q., Zhang Y., Song Y., Qi F., Zhai Q. (2007). Influence of ultrasonic vibration on mechanical properties and microstructure of 1Cr18Ni9Ti stainless steel. Mater. Des..

[17] Liu Q., Zhai Q., Qi F., Zhang Y. (2007). Effects of power ultrasonic treatment on microstructure and mechanical properties of T10 steel. Mater. Lett..

[18] Liu X., Osawa Y., Takamori S., Mukai T. (2008). Microstructure and mechanical properties of AZ91 alloy produced with ultrasonic vibration. Mater. Sci. Eng..

[19] Yao L., Hao H., Ji S., Fang C., Zhang X. (2011). Effects of ultrasonic vibration on solidification structure and properties of Mg-8Li-3Al alloy. Trans. Nonferrous Met. Soc. China.

